# Effects of Angiotensin Converting Enzyme Inhibitors on Liver Fibrosis in HIV and Hepatitis C Coinfection

**DOI:** 10.1155/2012/978790

**Published:** 2012-11-01

**Authors:** Lindsey J. Reese, Diane S. Tider, Alicia C. Stivala, Dawn A. Fishbein

**Affiliations:** ^1^Infectious Disease of Indiana, PSC, 11455 N. Meridian Street Suite 200, Carmel, IN 46032, USA; ^2^Department of Medicine, Mount Sinai School of Medicine, New York, NY 10029, USA; ^3^Jack Martin Coinfection Clinic, Mount Sinai Medical Center, One Gustave L. Levy Place, P.O. Box 1009, New York, NY 10029, USA; ^4^Department of Infectious Diseases, MedStar Washington Hospital Center, 110 Irving Street NW, Room 2A56, Washington, DC 20010, USA

## Abstract

*Background*. Liver fibrosis is accelerated in HIV and hepatitis C coinfection, mediated by profibrotic effects of angiotensin. The objective of this study was to determine if angiotensin converting enzyme inhibitors (ACE-Is) attenuate liver fibrosis in coinfection. *Methods*. A retrospective review of 156 coinfected subjects was conducted to analyze the association between exposure to ACE-Is and liver fibrosis. Noninvasive indices of liver fibrosis (APRI, FIB-4, Forns indices) were compared between subjects who had taken ACE-Is and controls who had not taken them. Linear regression was used to evaluate ACE-I use as an independent predictor of fibrosis. *Results*. Subjects taking ACE-Is for three years were no different than controls on the APRI and the FIB-4 but had significantly higher scores than controls on the Forns index, indicating more advanced fibrosis. The use of ACE-Is for three years remained independently associated with an elevated Forns score when adjusted for age, race, and HIV viral load (*P* < 0.001). There were significant associations between all of the indices and significant fibrosis, as determined clinically and radiologically. *Conclusions*. There was not a protective association between angiotensin inhibition and liver fibrosis in coinfection. These noninvasive indices may be useful for ruling out significant fibrosis in coinfection.

## 1. Introduction

With the advent of antiretroviral therapy (ART), the mortality from human immunodeficiency virus (HIV) has decreased; thus, other comorbidities, such as liver disease, have become increasingly important [1, 2]. End-stage liver disease, often from chronic hepatitis C (CHC), is currently one of the leading causes of death in HIV-positive persons [1, 3–5]. It is estimated that approximately 30% of HIV-positive persons are infected with hepatitis C virus (HCV), with estimates up to 50–90% in persons infected through intravenous drug use [6–9]. Moreover, liver fibrosis is accelerated in persons coinfected with HIV and HCV (“coinfection”) [10–15]. One study estimated that coinfected persons progress to cirrhosis in 7 years, on average, from time of HCV infection compared to 23 years in HIV-negative persons [10]. Thus, research is needed on mechanisms of fibrosis in liver disease from HCV and potential antifibrotic therapies, especially in coinfection. 

During liver injury, hepatic stellate cells become activated and produce elements of extracellular matrix leading to fibrosis [16, 17]. Stellate cell activation is stimulated by profibrotic cytokines, such as transforming growth factor-*β*1 (TGF-*β*1), and multiple studies have demonstrated the role of TGF-*β*1 in liver fibrosis [18–20]. Recent research has focused on the renin-angiotensin system as one mechanism of liver fibrosis, in that angiotensin II, the product of this system, has been shown to augment both TGF-*β*1 and stellate cells directly [21–26]. Infusion of angiotensin II increased TGF-*β*1 in bile-duct-ligated mice, accelerating liver fibrosis [27], and angiotensin receptor blockers (ARBs) have been shown to attenuate liver fibrosis by downregulating TGF-*β*1 production and other components of extracellular matrix that contribute to hepatic fibrogenesis [28–33]. In mouse models of nonalcoholic steatohepatitis (NASH), ARBs decreased activation of hepatic stellate cells and expression of TGF-*β*1, attenuating liver fibrosis and leading to improved survival [34–36]. Similar results have been seen with angiotensin converting enzyme inhibitors (ACE-Is), as enalapril was found to reduce TGF-*β*1 and other markers of extracellular matrix in bile-duct-ligated fibrotic mice [37]. 

In humans, ACE-Is and ARBs are used to slow the progression of fibrosis in diabetic nephropathy and heart failure [38–40]. There are few studies demonstrating their anti-fibrotic effects in chronic liver disease [41–44]. A small group of subjects with NASH had decreases in markers of hepatic fibrosis including TGF-*β*1, less activation of stellate cells, and histological improvement on liver biopsy after treatment with ARBs [41, 42]. In CHC, one study showed that subjects given ARBs had decreases in TGF-*β*1 concentrations and smaller areas of fibrosis when compared to subjects not treated with angiotensin blockade [43]. In a study of patients with CHC and hypertension, subjects receiving angiotensin-blocking agents had significantly less fibrosis than subjects who had not received these medications. These results suggested an association between liver fibrosis and hypertension, mediated via the renin-angiotensin system, and demonstrated a beneficial role of angiotensin blockade in CHC-related fibrosis [45]. Similarly, a recent prospective study compared 12 patients with CHC and hypertension who were treated with an ARB to those not receiving an ARB [46]. Those receiving an ARB showed significant improvements in liver fibrosis, as assessed by noninvasive indices of fibrosis. Last, a retrospective study in liver-transplant recipients with HCV recurrence showed that subjects receiving an ACE-I/ARB had less graft fibrosis [44]. To our knowledge, no studies have examined the use of angiotensin inhibition in coinfection, which is a population with more rapidly progressive liver disease. 

We hypothesized that subjects treated with angiotensin blockade therapeutics would have decreased levels of fibrosis as measured by noninvasive indices of fibrosis, including the aspartate aminotransferase to platelet ratio index (APRI) [47], the FIB-4 index [48, 49], and the Forns score [50]. These indices have been validated in the literature as noninvasive indices of liver fibrosis for use in HCV and in coinfection [51–54]. We retrospectively examined the potential anti-fibrotic effects of angiotensin blockade in subjects coinfected with HIV and HCV.

## 2. Materials and Methods

### 2.1. Patient Population

This is a retrospective chart review of a cohort of 199 subjects with HIV/HCV coinfection in the Mount Sinai Medical Center HIV Clinic from June 2006 to October 2007. Men and women who were at least 18 years old were included if they had documented infection with both HIV and HCV. Subjects were excluded if they were actively undergoing treatment for HCV or if they had a positive HCV antibody but no detectable viral load; 156 patients were included in the final analysis. The protocol was approved by the Institutional Review Board of the Mount Sinai School of Medicine, and all work was conducted in accordance with the Declaration of Helsinki.

### 2.2. Study Procedures

Using written and computerized chart data over three years, the following information was abstracted for each subject: age, gender, race, ethnicity, body mass index (BMI) (kg/cm^2^), current medications, CD_4_ lymphocyte count (cells/mL), HIV viral load (copies/mL), quantitative HCV RNA (IU/mL), alcohol use, diabetic indicators (hemoglobin A_1_C, degree of proteinuria, and measures of preserved blood glucose), liver biopsy results (using the Scheuer score [55] of 4 as indicative of severe fibrosis), radiographic studies commenting on the degree of liver injury or indicators of advanced fibrosis (such as varices, ascites, and/or splenomegaly), and laboratory data used to compute the noninvasive indices, as described below. Biopsy data, when available, was combined with radiologic findings to make a clinical determination of the extent of liver disease. This clinical data was then used to validate the noninvasive indices of fibrosis (see Section 3). Using a computerized medical database, serological data including platelets, aspartate aminotransferase (AST), alanine aminotransferase (ALT), albumin, gamma-glutamyl transferase (GGT), and total cholesterol were collected for all subjects at time of data collection, one year prior to that time and three years prior to data collection. As this was a retrospective chart review, all data (current, one year prior, and three years prior) was collected at one point in time. These values were used to determine the APRI [47], the FIB-4 index [48, 49], and the Forns score [50]. Each of the indices has cut-off scores, predetermined and validated in HCV monoinfected persons (and in coinfected persons for the FIB-4 index) that are predictive of significant fibrosis: for the APRI, a score greater than 1.5 has a positive predictive value (PPV) of 91% for significant fibrosis (Ishak fibrosis score [56] >4) [47]; for the FIB-4, a score greater than 3.25 has a PPV of 65% for significant fibrosis (Ishak fibrosis score >3) [48]; for the Forns, a score greater than 6.9 has a PPV of 79% for significant fibrosis (Scheuer's classification stage >2) [50]. We reexamined these indices in our study population as well and found them to be valid in measuring significant fibrosis (see Section 3). 

### 2.3. Statistical Analysis

 In order to compare the univariate analysis of baseline characteristics, chi-square and Fisher's exact tests were used for categorical data; Student's *t*- and Mann-Whitney tests were used for continuous, normally distributed and nonparametric data, respectively. The noninvasive indices were compared across three groups: one group had taken an ACE-I or ARB for one year, one group for up to three years, and one group had not taken these medications, referred to as the control group. For each comparison, the three noninvasive indices were analyzed as dichotomous variables using the chi-square analysis and the predefined cut-off values, and as continuous variables. Linear regression models were used to identify factors independently associated with the aforementioned outcomes. Variables with *Pvalues *<0.25 on a univariate basis or that were biologically plausible were included in the multivariate models. SPSS version 16 software was used for the analysis. 

## 3. Results

### 3.1. Study Sample

Baseline characteristics of the 156 subjects are shown in Table 1. Forty-two subjects (26.9%) had an exposure to an ACE-I/ARB: 33 (21.2%) had taken an ACE-I/ARB for at least one year and 23 (14.7%) had taken an ACE-I/ARB for three years. The majority of subjects were taking an ACE-I (lisinopril, enalapril, quinapril, or fosinopril); one subject was taking losartan. Subjects in the control group were younger and were significantly less likely to be black compared to the group exposed to an ACE-I/ARB. The median HIV viral load in subjects who had detectable viremia was significantly higher in the control group compared to the group exposed to an ACE-I/ARB, but the percentage of subjects with an undetectable viral load was similar between the groups (52.6% versus 66.7%, resp.) as were the median CD_4_ cell counts. There was no difference between the groups in alcohol use or in prior treatment with interferon, and the median HCV RNA was similar between the groups. Forty-nine subjects (31%) had biopsy data; there were no paired or serial biopsies. Fifty subjects (32%) had severe fibrosis by biopsy or had radiological indicators of advanced fibrosis; this was not significantly different between the groups. The groups did differ significantly on prevalence of diabetes and other diabetic indicators such as proteinuria and levels of hemoglobin A_1_C; subjects in the control group were significantly less likely to have diabetes than subjects taking an ACE-I/ARB. 

### 3.2. Noninvasive Indices

There were significant associations between the aforementioned cut-off values predictive of significant fibrosis for all indices and clinical evidence of advanced fibrosis (biopsy data and radiological indicators of advanced liver disease) (Table 2). In the present study, when compared to clinical indicators of fibrosis, an APRI score of less than 0.5 had a negative predictive value (NPV) to exclude any fibrosis of 86.9% (*P* < .001); a score greater than 1.5 had a PPV for significant fibrosis of 68.4% (*P* < .001). This is compared to an NPV of 86% and a PPV of 88% based on the original literature in a monoinfected population [47]. The prevalence of advanced fibrosis in the original study was 47% compared to 32% prevalence in this study, possibly accounting for the higher PPV in the original study. A FIB-4 score of less than 1.45 had an NPV of 89.7% for excluding any fibrosis (*P* = .002), and a score greater than 3.25 had a PPV of 62.7% (*P* < .001). These results were similar to the original literature, an NPV of 90% and a PPV of 65% [48]. A Forns score less than 4.2 had an NPV for excluding fibrosis of 100% in this sample (*P* = .01), a score greater than 6.9 had a PPV of 64.1% (*P* < .001). These results were similar to the original literature, an NPV of 96% and a PPV of 66% [50].

### 3.3. Levels of Fibrosis

On univariate analysis, fibrosis scores for subjects taking ACE-Is/ARBs for less than three years, but at least one year, were no different than scores for the control group on any of the indices (Tables 3-4). Fibrosis scores for subjects taking ACE-Is/ARBs for at least three years were no different than scores for the controls on the APRI and FIB-4 (Tables 3-4) but were significantly higher than scores for the controls on the Forns index when analyzed as continuous variables using a Student's *t*-test (9.31 and 6.83 resp., *P* < 0.001) (Table 3). On chi-square analysis, all 12 subjects (100%) in the ACE-I/ARB group had a Forns score greater than 6.9, indicative of significant fibrosis, versus 47.4% in the control group (*P* < 0.001) (Table 4).

These results were verified in multivariate analysis (Table 5). Factors predicting higher Forns scores (68 observations) included ACE-I/ARB use for three years, clinical evidence of advanced fibrosis, older age, black race, and lower HIV viral load. The use of ACE-I/ARBs for at least three years was independently associated with an elevated Forns score when adjusted for the aforementioned variables (*P* < 0.001). The combination of the above variables created a significant linear regression model (*P* < 0.001) with the highest adjusted *R* ^2^ (0.587) and the lowest standard error (1.50). Table 5 lists the variables associated with an elevated Forns score along with the additional variables that were rejected from the model. There were no significant interactions found between any of the variables in the final model. 

## 4. Discussion

We hypothesized that subjects treated with angiotensin blockers would have decreased levels of fibrosis as measured by noninvasive indices when compared to subjects not exposed to these medications. Conversely, we found that angiotensin blockade in a cohort of coinfected subjects did not attenuate the progression of liver fibrosis. In fact, there was a statistically significant correlation of worsening fibrosis on the Forns index for subjects who had taken ACE-Is/ARBs for three years compared to subjects who had not been exposed to these medications. While not statistically significant, the trend in the ACE-I/ARB group was progressively worse in all groups at one year and continued to worsen when going back three years. This finding is in contrast to prior data that portrayed an anti-fibrotic effect of angiotensin inhibition. To our knowledge, though, this is the only study that has looked at angiotensin inhibition in subjects with coinfection. 

 There are a few plausible explanations for our conflicting results. First, we did not distinguish between subjects who were taking an ARB or an ACE-I. A recent study in bile-duct-ligated mice suggests that ARBs may be more effective in suppressing hepatic fibrosis compared to ACE-Is [57]. A future study may show different results depending on the method of angiotensin suppression. In addition, there was no standardization of dose of ACE-I or ARB. It is possible that high doses of these medications could lower blood pressure enough to impair liver perfusion causing worsening of fibrosis scores. Doses of these medications should be standardized in future research. 

Another possible explanation is that subjects taking an ACE-I or ARB had more unmeasured comorbidities than subjects not on these medications. The ACE-I or ARB may have been added for HIV-associated or diabetic nephropathy, hypertension, or heart failure. It is possible that these subjects appeared to have elevated fibrosis scores because they were sicker than the group that did not require these medications. On multivariate analysis, though, ACE-I/ARB use was independently associated with an elevated Forns score, after controlling for co-morbidities such as diabetes. In addition, older subjects and subjects of black race had significantly higher Forns scores, which is consistent with data from prior studies that have shown worsening disease and poorer treatment responses in these groups [58, 59]. Subjects in the control group were younger and were significantly less likely to be black when compared to the ACE-I/ARB group, which could have created a healthier control group. 

There also could be a deleterious interaction between HIV positivity and angiotensin blockade or between ART for HIV and ACE-Is/ARBs. On multivariate analysis, lower levels of HIV viral load were associated with elevated Forns scores. It is possible that subjects who had lower HIV viral loads were taking ART and had medication-induced liver toxicity either from the combination of ACE-Is/ARBs and ART or from ART alone. This explanation could be examined by comparing fibrosis indices in a similar cohort of subjects with HCV monoinfection who have been treated with an ACE-I or ARB. 

A recent study in CHC monoinfection relates the possibility that the effects of angiotensin inhibition may occur earlier in liver fibrosis and may be missed in persons with advanced liver disease, often seen in coinfection [60]. This study used data from the Hepatitis C Long-term Treatment against Cirrhosis (HALT-C) Trial [61] to evaluate the effects of continuous ACE-I/ARB use for 3.5 years on liver fibrosis progression as determined by serial liver biopsies [60]. Researchers were unable to demonstrate a benefit for angiotensin blockade in attenuating progression of liver fibrosis: 33.3% of subjects on an ACE-I/ARB had a 2-point increase in fibrosis, compared to 32.5% and 25.7% of subjects on other antihypertensives or on no medications, respectively (*P* = 0.21). The authors comment that the majority of their subjects had significant fibrosis at baseline, which is similar to our coinfected cohort. If angiotensin inhibitors exert maximal antifibrogenic effects early in the fibrosis process and have decreased activity at later stages of fibrosis, the affects of these medications may be missed in a cohort with advanced liver disease. This may especially be true in coinfection as the progression of liver injury is often more rapid than in HCV monoinfection; thus, it would be prudent to determine the anti-fibrotic nature of these medications soon after diagnosis of HCV, before the development of liver fibrosis. 

One reason for the limited number of human studies in liver fibrosis is the need for invasive measurements, such as liver biopsy, to record the progression of liver disease. Moreover, liver biopsy has been shown to be a less than ideal gold standard for comparison. Recent research has focused on noninvasive indices of liver fibrosis, using common laboratory values to estimate the severity of liver disease. Three of these indices, the Forns score, the APRI, and the FIB-4, were used and validated in the present study to document liver fibrosis in subjects with coinfection. We found a correlation between the presence or lack of significant fibrosis as determined by radiological and biopsy data and level of fibrosis as determined by the use of these noninvasive indices in coinfected subjects. The PPVs were too low to be considered clinically useful, but the NPVs for all of the indices were high enough to make them clinically relevant in ruling out significant fibrosis. This is important as these indices may be used to identify persons at low risk for significant fibrosis and possibly decrease the need for liver biopsies in this group. At more advanced levels of immunosuppression from HIV it seems possible that some of these markers would be susceptible to error, such as from HIV-associated thrombocytopenia. The present study validated these indices despite a range of CD_4_ counts and HIV viral loads. The best use of these indices in a coinfected population would be to rule out significant fibrosis and avoid biopsy, possibly in a patient who is delaying treatment and has no other signs of hepatic inflammation. 

 The current study has limitations that prevent definitive conclusions on the effects of angiotensin inhibition. Due to the retrospective nature of the study, causation cannot be inferred. Prospective and ideally randomized controlled trials are needed to determine if these medications may have hepatic toxicity alone, or in combination with ART. Also, fibrosis scores became higher overtime, as cumulative exposure to an ACE-I or ARB was increasing. There were a small number of subjects on ACE-Is/ARBs consistently over the years, though, which may have limited our power to detect a significant difference between the groups earlier than three years. To verify this trend, further research should follow a larger number of subjects over a longer period of time, including the time preceding significant liver fibrosis. Last, we did not have data on other mechanisms of fibrosis, such as non-alcoholic fatty liver disease (NAFLD). Although we collected data on body mass index and diabetes, we did not always have a biopsy-proven diagnosis of NAFLD, which may result in higher fibrosis scores despite being on an ACE-I or ARB. 

## 5. Conclusions

 In conclusion, in the present study, we validated three noninvasive indices of fibrosis in an HIV/HCV coinfected cohort. As liver biopsy is not without risk, it may be useful to have noninvasive methods to rule out advanced liver disease in coinfection. We did not find a beneficial effect of angiotensin blockade in these subjects, contradicting prior studies in HCV monoinfection. Of concern was that subjects taking an ACE-I or ARB had higher levels of fibrosis than controls after three years on one of the indices. Despite the aforementioned limitations, treatment with an ACE-I or ARB to slow liver fibrosis in coinfection requires further study. There could be a deleterious interaction between HIV positivity and angiotensin blockade that could be examined by comparing fibrosis indices in a cohort of subjects with HCV monoinfection who have been exposed to these medications.

## Figures and Tables

**Table 1:** Baseline characteristics of 156 subjects characterized by ACE-I/ARB exposure.

Variable	No ACE-I/ARB (*n* = 114)	ACE-I/ARB (*n* = 42)	*P* value
Mean age (years) ± standard deviation (SD)	50.3 ± 8.6	53.4 ± 5.5	**0.04**
Male gender (%)	76 (66.7)	31 (73.8)	0.40
Black race (%)	34 (29.8)	20 (47.6)	**0.04**
Hispanic ethnicity (%)	67 (58.8)	21 (50)	0.33
Mean BMI (kg/m^2^) ± SD	25.6 ± 6.2	27.0 ± 6.8	0.34
Median HCV RNA (IU/mL) (range)	3.66 × 10^6^ (600–4.23 × 10^7^)	3.54 × 10^6^ (600–3.08 × 10^7^)	0.70
Median log_10_ HCV RNA (range)	6.56 (2.78–7.63)	6.55 (2.78–7.49)	0.70
Undetectable HIV viral load (%)	60 (52.6)	28 (66.7)	0.12
Median HIV viral load* (copies/mL) (range)	1.61 × 10^4^ (58–8.21 × 10^5^)	421 (65–2.11 × 10^5^)	**0.03**
Median log_10_ HIV viral load* (range)	4.21 (1.76–5.91)	2.62 (1.81–5.33)	**0.03**
Median CD_4_ cell count (cells/mL) (range)	372 (11–2193)	389 (51–1183)	0.31
Alcohol Use (%)	37 (32.5)	9 (21.4)	0.18
Prior HCV Treatment (%)	8 (5.1)	6 (3.8)	0.16
Diagnosis of diabetes (%)	13 (11.4)	23 (54.8)	**<0.001**
Proteinuria (%)	34 (32.1)	25 (61)	**<0.001**
Mean hemoglobin A1C (g/dL) ± SD	5.37 ± 0.66	6.15 ± 1.33	**0.03**
Significant fibrosis by clinical data (%)	32 (28.1)	18 (42.9)	0.08

^∗^Calculated in those with detectable HIV viral load.

**Table 2:** Accuracy of the non-invasive indices in predicting significant fibrosis.

Indices	Fibrosis by clinical data (%)	Sensitivity %	Specificity %	PPV %	NPV %
APRI					
<0.50	8 (16)	84	50	44	87
>0.50	42 (84)				
<1.50	20 (48)	52	89	68	79
>1.50	26 (52)				

FIB-4					
<1.45	4 (8)	92	33	40	90
>1.45	46 (92)				
<3.25	13 (26)	74	79	63	86
>3.25	37 (74)				

Forns					
<4.21	29 (100)	100	20	48	100
>4.21	0 (0)				
<6.90	4 (14)	86	65	64	87
>6.90	25 (86)				

**Table 3:** Comparison of continuous fibrosis scores between groups at one year and three years (univariate analysis).

Indices	Control group	ACE-I/ARB group	*P* value
Mean APRI scores at one year ± SD	1.16 ± 1.42	1.30 ± 1.43	0.76
Mean APRI scores at three years ± SD	1.17 ± 1.40	1.33 ± 1.57	0.87
Mean FIB4 scores at one year ± SD	3.57 ± 3.49	4.21 ± 4.02	0.36
Mean FIB4 scores at three years ± SD	3.53 ± 3.36	4.72 ± 4.72	0.38
Mean Forns scores at one year ± SD	7.01 ± 2.43	7.93 ± 1.96	0.14
Mean Forns scores at three years ± SD	6.83 ± 2.27	9.31 ± 1.36	**<0.001**

**Table 4:** Number of subjects from each group with scores predictive of significant fibrosis at one year and three years (univariate analysis).

Indices	Control group	ACE-I/ARB group	Odds ratio (95% confidence interval)*	*P* value
APRI score > 1.5 at one year (%)	28 (23)	10 (30)	0.76 (0.41–1.40)	0.39
APRI score > 1.5 at three years (%)	31 (24)	7 (30)	0.77 (0.39–1.54)	0.48
FIB4 score > 3.25 at one year (%)	43 (35)	16 (49)	0.73 (0.48–1.11)	0.17
FIB4 score > 3.25 at three years (%)	48 (36)	11 (48)	0.76 (0.47–1.23)	0.30
Forns score > 6.9 at one year (%)	25 (50)	14 (74)	0.68 (0.46–1.00)	0.08
Forns score > 6.9 at three years (%)	27 (47)	12 (100)	0.47 (0.36–0.62)	**<0.001**

^∗^Odds ratio for an elevated score in the control group compared to the ACE-I/ARB group.

**Table 5:** Factors associated with an elevated Forns score on multivariate analysis (*n* = 69)*.

Variable	Beta estimate**	Standard error	*P* value
ACEI use for three years	1.924	0.507	**<0.001**
Age	0.060	0.024	**0.014**
Race	−1.174	0.395	**0.004**
HIV viral load	−5.893e^−6^	0.000	**0.02**
Clinical evidence of cirrhosis	2.129	0.380	**<0.001**
Gender	0.304	0.386	0.434
BMI	−0.017	0.032	0.584
Diabetes	−0.500	0.432	0.251
Proteinuria	−0.003	0.002	0.155
HCV viral load	−2.055e^−8^	0.000	0.397
HBV surface antigen positivity	−1.285	1.153	0.27
Prior treatment with interferon	0.444	0.706	0.532
Alcohol use	0.332	0.425	0.437
Marijuana use	−0.186	0.898	0.836

*The final model included ACEI use for three years, age, race, HIV viral load, clinical evidence of cirrhosis with an adjusted *R* ^2^ of 0.587, a standard error of the estimate of 1.498, and an overall *P* value < 0.001.

**Beta estimate is the magnitude of effect that each variable has on the Forns score.
